# Dissociation of place preference and tolerance responses to sucrose using a dopamine antagonist in the planarian

**DOI:** 10.1007/s00213-017-4801-8

**Published:** 2017-12-02

**Authors:** Rafat A. Mohammed Jawad, Claire V. Hutchinson, Jose Prados

**Affiliations:** 1 Department of Neuroscience, Psychology and Behaviour, University of Leicester, University Road, Leicester, LE1 7RH UK; 2 Muthanna University, Samawah, Iraq

**Keywords:** Planarian, Sucrose addiction, Conditioned place preference, Tolerance, Dopamine reward system

## Abstract

In rodents, sucrose has been found to elicit addictive-like behaviours like the development of tolerance and the association with cues present at the time of consumption. Furthermore, the neurochemical response to sucrose binges is equivalent to the one observed in response to the abuse of addictive substances like cocaine. The experiments reported here address the effects of sucrose on an invertebrate model, the Platyhelminth brown planarian. The animals exposed to a 10% sucrose solution in one context developed a conditioned place preference (CPP) which was subsequently extinguished in the absence of the rewarding agent. However, one exposure to sucrose per se sufficed to *reinstate* the CPP response, suggesting sucrose-induced CPP can be characterised as a standard Pavlovian response. The same training procedure led to the development of context-specific tolerance to the effects of sucrose. However, comparing animals treated with dopamine D1 antagonist (SCH-23390) with control animals showed that the establishment of CPP, but not the development of tolerance, is mediated by the dopamine reward system.

## Introduction

Following continuous consumption of sweet foods containing sucrose, deprivation can induce  in some individuals addictive-like behaviours that lead to increased sweet food intake, a harmful cycle that might contribute to obesity and diabetes (Gearhardt et al. 2011). Sucrose has been characterised as a substance of abuse in animal models: rats exposed to sucrose display a number of behavioural and physiological responses similar to those elicited by drugs of abuse like cocaine or amphetamine (Avena et al. 2008). Those include behavioural changes like conditioned place preference (CPP), withdrawal and craving (Avena et al. 2005; Wideman et al. 2005), and physiological responses like enhancement of extracellular dopamine in the nucleus accumbens (Bassareo and Di Chiara 1997; Rada et al. 2005).

Addictive behaviours have been described as an instance of learned Pavlovian conditioned responses. Pairing a particular set of contextual cues with the experience of a rewarding drug typically results in the development of CPP. This experience is also likely to result in the development of a second conditioned response which tends to reduce the effects of the drug: the post-intake effects in a naïve individual are opposed by an innate compensatory response that can be controlled by the contextual cues (Remington et al. 1997; Siegel 1975). The development of conditioned compensatory responses to the cues associated with drug usage elegantly account for the development of tolerance. Furthermore, prompting of the conditioned compensatory response leads to an imbalance that can be restored by consumption of the drug. If the drug is not available, the animal will experience distress and show symptoms of withdrawal and drug-seeking behaviour.

Although the development of CPP can be assumed to depend on the rewarding properties of the sucrose-unconditioned stimulus (US) modulated by the dopamine reward system, the development of a conditioned compensatory response (which is not necessarily a pleasant one) might be independent of this reward system. To assess this hypothesis, we developed CPP and tolerance training procedures using an invertebrate model, the Platyhelminth brown planarian, and compared the conditioned responses developed by the animals treated with dopamine D1 antagonist with control animals.

*Planaria* offer a good pre-clinical model for substance abuse. Their nervous system presents structural and physiological similarities to the nervous system of vertebrates: centralised and bilateral with similar neural networks, transmitters, and neuromodulators (Buttarelli et al. 2008; Inoue et al. 2015). They also exhibit complex learning in standard Pavlovian and instrumental conditioning tasks (e.g. Lee 1963; Prados et al. 2013) and display behavioural responses to drugs of abuse that are similar to those seen in mammals, including cocaine behavioural sensitization (Rawls et al. 2010) and CPP (Amaning-Kwarteng et al. 2017; Hutchinson et al. 2015).

In our study, we aimed to characterise CPP and tolerance development as examples of Pavlovian conditioned responses (CRs). One key feature of Pavlovian CRs is that they are subject to extinction. Therefore, we assessed the acquisition and extinction of CPP responses; the development of conditioned compensatory responses during the course of tolerance training, and, finally, we compared the effect of blocking the D1 dopamine receptors on CPP and tolerance.

## Methods

### Subjects

Ninety six brown *Planaria* (*Dugesia*) purchased from Blades Biological Ltd. (Kent, UK) served as the subjects in the present study. The flatworms were held in a plastic container filled with two litres of water treated with 1 ml/l Aquasafe (Aquasafe, Tetra, Germany). The planarian colony was kept at a room temperature of 20 °C (± 2) with a light cycle of 14/10 h. The animals were fed raw chicken meat daily for 1–2 h; the water of the aquarium was changed daily after feeding the animals; they were deprived of food, however, from 2 days before the start of the experiments.

### Materials

The animals were exposed, during the experimental sessions, to plastic dishes (9 cm diameter) which could have a smooth surface (plain plastic), a rough surface (white sand glued to the dish using transparent silicone), or one half smooth and one half rough. Throughout the experiments, the animals could be exposed to treated water, a 10% sucrose solution, a 1-μM solution of a selective D1 dopamine receptor antagonist^1^ (SCH-23390 hydrochloride, Sigma-Aldrich, UK), or a mixture of 10% sucrose and a 1-μM SCH-23390 solution. During the experimental sessions, the animals’ activity was tracked by using a Video-Track System (ViewPoint, France).

### Procedures

We report three experiments. The first one addressed the acquisition and extinction of CPP. This experiment used sucrose as the rewarding agent and compared the development and extinction of CPP in animals treated with a dopamine antagonist and in a control non-treated group. Experiment 2 assessed the development of conditioned compensatory responses to sucrose. Finally, experiment 3 compared the effect of the dopamine antagonist in the development of CPP and the conditioned compensatory responses.

#### Experiment 1: conditioned place preference (CPP)

**Fig. 1:**
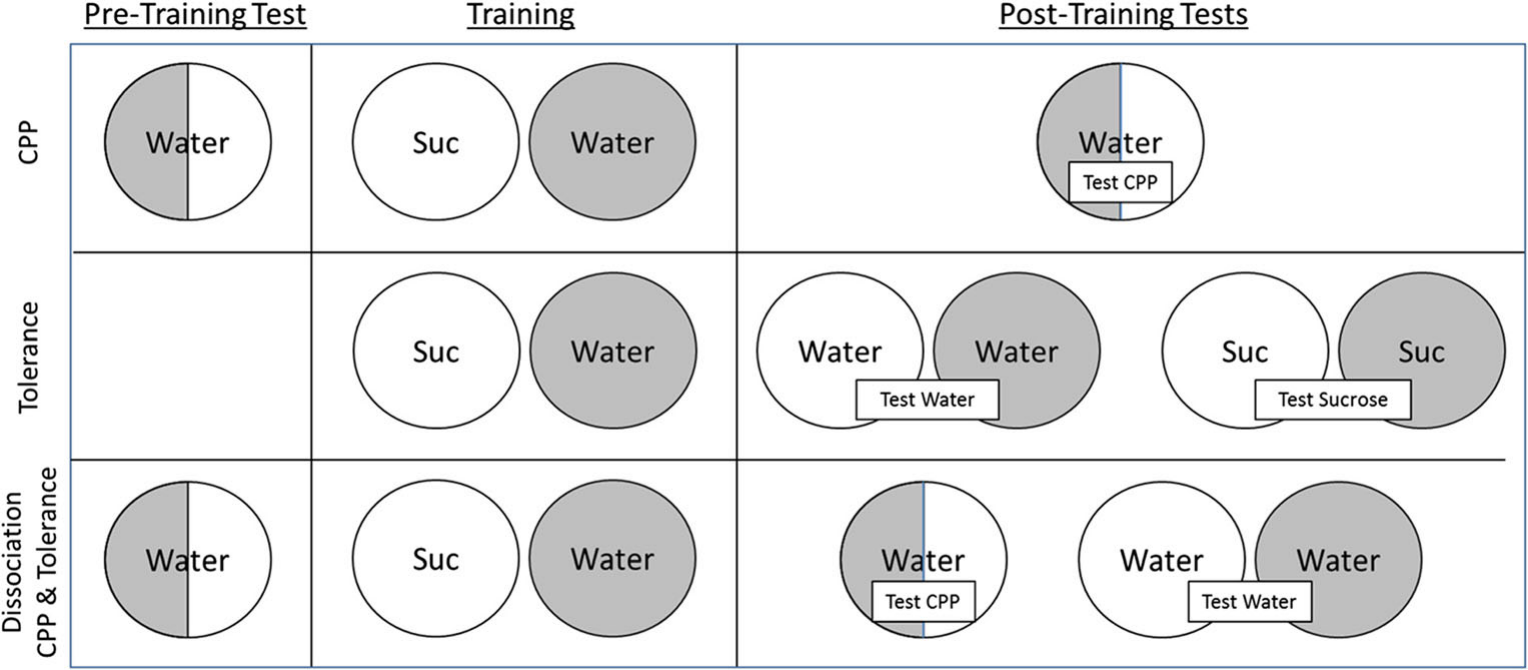
Schematic representation of the procedure of the experiments: top row, conditioned place preference; middle row, tolerance as the development of a conditioned compensatory response; lower row, dissociation between CPP and tolerance. The white and shadowed areas represent the two surfaces used in the experiments (plastic and sand textures). Sucrose refers to the presentation of a 10% sucrose solution in the trained context (in CPP experiments, the less preferred context during the pre-training test)

The procedure involved three phases: pre-training test (day 1 of experiment), training (days 2–9), and post-training test (days 10–14; see Fig. 1, top row). For the test trials (pre- and post-training), the animals were exposed to treated water in one of the two-sided (half smooth—half rough) petri dishes. They were allowed to freely move for 30 min; the time spent in each of the two surfaces of the petri dish was recorded, and a preference score was calculated for the less preferred surface (time spent in the less preferred surface/total time).

The training phase took place after the pre-training test and lasted 8 days in which the animals were exposed to the two surfaces in alternation every 24 h. The animals were exposed for 30 min to a 10% sucrose solution^2^ in the less preferred surface during, for example, the odd days, and to treated water for 30 min in the preferred surface in the even days; this cycle was repeated four times. They were assigned to one of two experimental conditions: the group D-Ant (*n* = 24), treated with the dopamine antagonist during all the sessions of the training phase, and the control group (*n* = 24) that was never presented with the dopamine antagonist. During the post-training tests, the animals were again exposed to the two-sided petri dishes. To establish whether the animals showed conditioned place preference, indicated by a shift of preferences in relation to the pre-training test, we subtracted the preference score observed for the less preferred context in the post-training test from the preference score observed during the pre-training test. A difference score of 0 would indicate no change in preferences, any positive value would indicate a change in the preferences (a CPP response). The animals were given four post-training test trials (days 10–13 of the experiment) to monitor the extinction of any CPP response observed (CPP extinction tests). Once the extinction of the CPP was completed, all the animas were re-exposed for 30 min to the sucrose solution in a distinct glass petri dish (5 cm in diameter) 1 hour after the last extinction trial (day 13). The following day, the animals were tested again in the two-sided petri dishes to assess the reinstatement of the CPP response (CPP reinstatement test).

#### Experiment 2: tolerance as the development of a conditioned compensatory response

For the tolerance experiments, we monitored the activity of the animals during the experimental sessions. It has been well established in our laboratory that exposure to sucrose reduces the motor activity of the *Planaria*. We hypothesised that as a result of repeated exposures to sucrose, the animals might develop a conditioned compensatory response of increased motor activity. Sixteen animals were given tolerance training by repeatedly presenting them with sucrose in a particular context; the animals were also exposed in alternating days to a different context in the presence of water. The procedure was, therefore, very similar to the CPP training phase described above. During the first 8 days of the experiment, half of the animals were exposed to sucrose in the smooth context for 30 min in the odd days and water in the rough context in the even days; for the other half of the animals, this arrangement was reversed. We refer to the context paired with sucrose as the trained context, and the one paired with water as the control context. The tolerance training phase was followed by 2 cycles of test trials in which the animals were tested in the trained and control contexts under two conditions: in the presence of water (test water, days 9 and 10 of the experiment) and in the presence of sucrose (test sucrose, days 13 and 14; see Fig. 1 middle row). The order in which the two surfaces were presented during each test was counterbalanced across subjects. On the days 11 and 12, the animals were given a re-training cycle in which they were re-exposed to sucrose in the trained context and water in the control context. The test water assessed the development of hyperactivity conditioned compensatory responses in the trained context. The test sucrose assessed the effectiveness of sucrose in reducing the activity of the animals in the trained and the control contexts.

#### Experiment 3: dissociation between CPP and tolerance

The animals were assigned at random to one of two groups: the control group (*n* = 16) and the D-Ant (*n* = 16) which was treated with dopamine antagonist during the training. There were three phases in the experiment: pre-training, training, and test (see Fig. 1, lower row). The pre-training and training phases (pre-training, day 1; training, days 2–13 of the experiment) followed the same procedure described for CPP above; the only change is that the animals were given 6 instead of 4 cycles of training. After the completion of the training phase, all the animals were given one CPP test (for example on day 14) and one test water to assess the development of the hyperactivity conditioned compensatory response (days 15–16). The order of the tests and the order in which the animals were exposed to the trained and control contexts during the test water were counterbalanced across subjects.

## Results

### Experiment 1: conditioned place preference (CPP)

The experiment assessed the acquisition and extinction of CPP and the role of the dopamine reward system. During the training phase, we recorded the levels of activity (distance covered during the 30-min session). The animals in the control group showed lower levels of activity in the presence of sucrose than in the presence of water. The animals in the D-Ant group showed lower levels of activity than the control group, and also, lower levels of activity in the presence of sucrose and the dopamine antagonist than in the presence of the dopamine antagonist alone. The animals in the control group covered a mean distance of 281 cm (± 7.73 *SEM*) in the trials in which they were exposed to sucrose in the less preferred context, and 376 cm (± 23.17) in the trials in which they were exposed to water in the preferred context. The animals in the D-Ant group covered a mean distance of 256 cm (± 7.11) in the presence of sucrose and the dopamine antagonist (in the less preferred context), and 317 cm (± 9.66) in the presence of the dopamine antagonist (in the preferred context). An ANOVA with group (control vs. D-Ant) and stimulus (sucrose vs. water) showed a significant effect of group (*F*(1.46) = 10.44, *p*<0.01, *η*_*p*_^2^ = .18) and stimulus (*F*(1.46) = 30.70, *p* < 0.01, *η*_*p*_^2^ = .40). The interaction between these factors was non-significant.

During the pre-training test, the animals in group control showed a preference score (for the less preferred context) of 0.34 (± 0.04 *SEM*); the animals in group D-Ant showed a preference score of 0.43 (± 0.02). A one-way ANOVA showed that there were no differences between the groups (*F*(1.46) = 3.53).

**Fig. 2:**
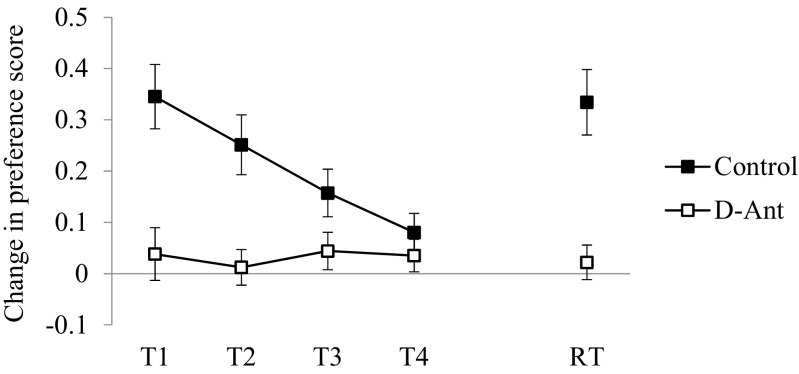
Mean change in preference score (± *SEM*) to the target context during the extinction (T1-T4) and reinstatement test (RT) trials of the test phase of experiment 1

The data for the CPP extinction tests and reinstatement test corresponding to the change in preference scores for the initially non-preferred context are displayed in Fig. 2. Only the animals in the control group developed a significant CPP response. An ANOVA with groups (control and D-Ant) and test trials (T1-T4) showed a significant effect of group (*F*(1.46) = 12.27, *p* < 0.01, *η*_*p*_^2^ = .21) and test trials (*F*(3.138) = 5.45, *p* < 0.01, *η*_*p*_^2^ = .10). The interaction groups x test trials was also significant (*F*(3.138) = 6.03, *p* < 0.01, *η*_*p*_^2^ = .11). Further analyses carried out to analyse this interaction showed that the animals in the group control showed a significant decrease in change of preference score over the 4 days of test (*F*(3.69) = 11.34, *p* < 0.01, *η*_*p*_^2^ = .33), whereas the animals in the D-Ant group showed no significant changes over the test trials, *F* < 1. Also, the two groups differed in tests trials 1 and 2 (*F*_*s*_(1.46) ≥ 12.35, *p*s < 0.01), but they did not differ in the tests 3 and 4. The data of the reinstatement test (RT) are displayed on the right hand part of Fig. 2. The animals in the control group showed a significant recovery of the CPP response after been exposed to the sucrose in a different context. A one-way ANOVA confirmed that the difference between the groups was significant (*F*(1.46) = 18.74, *p* < 0.01).

### Experiment 2: tolerance as the development of a conditioned compensatory response

The previous experiment showed that exposure to sucrose decreases the planarian’s motor activity. In the present experiment, we monitored the hypoactivity unconditioned response to sucrose to assess whether the animals develop tolerance to this substance over repeated exposures. The hypothesis under this test was whether the animals would develop a conditioned compensatory response of hyperactivity that would be controlled by the contextual cues associated with sucrose.

**Fig. 3:**
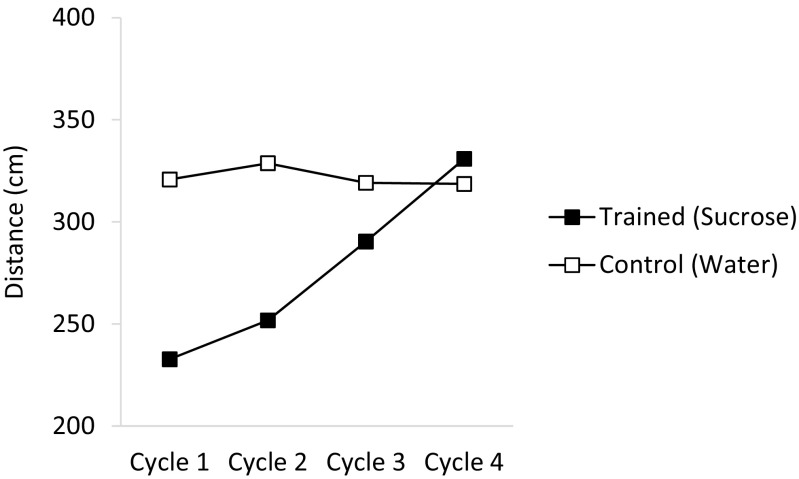
Mean distance covered by the animals in the contexts were sucrose (trained context) and water (control context) were presented over 4 cycles of training during the tolerance training phase of experiment 2

The data of the tolerance training phase of the experiment are displayed in Fig. 3. The animals showed lower levels of activity in the presence of sucrose than in the presence of water during the first 2 cycles of training. However, the activity of the animals in the presence of sucrose gradually increased over the training days. A within-subject ANOVA with stimulus (sucrose vs. water) and training cycles showed significant effects of stimulus (*F*(1.15) = 18.84, *p* < 0.01, *η*_*p*_^2^ = .55); training cycles (*F*(3.45) = 5.22, *p* < 0.01, *η*_*p*_^2^ = .25); and a significant interaction stimulus x training cycles (*F*(3.45) = 6.281, *p* < 0.01, *η*_*p*_^2^ = .29). Further analyses carried out to analyse this interaction showed that animals showed a significant increase in the levels of activity over the four training trials in which they were exposed to sucrose (*F*(3.45) = 18.08, *p* < 0.01, *η*_*p*_^2^ = .54), whereas their levels of activity in the trials in which they were exposed to treated water remained unchanged, *F* < 1. Also, the animals showed less activity in sucrose than in water in the training cycles 1 and 2 (*F*_*s*_(1.15) ≥ 18.434, *p*s < 0.01); in the cycles 3 and 4, however, the levels of activity in sucrose and water did not differ.

During the re-training cycle (carried out between the test water and the test sucrose), the animals showed lower activity levels when exposed to sucrose (301.70 cm, ± 12.12 *SEM*) than when exposed to water (342.96 cm, ± 10.22) (*F*(1.15) = 4.73, *p* < 0.05, *η*_*p*_^2^ = .24).

**Fig. 4:**
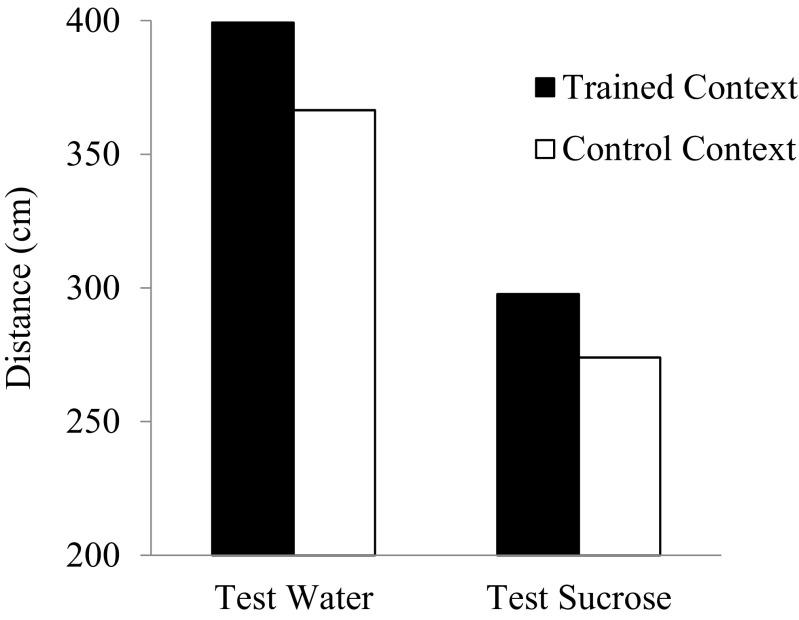
Mean distance covered by the animals in the trained and the control contexts throughout the 2 cycles of test trials in the presence of water and sucrose in experiment 2

The data of the test phase of the experiment are displayed in Fig. 4. Overall, the animals showed higher levels of activity in the trained than in the control context. In addition, the animals showed higher levels of activity in the test water than in the test sucrose. A within-subject ANOVA with stimulus (sucrose vs. water) and context (trained vs. control) confirmed these impressions, showing a significant effect of stimulus (*F*(1.15) = 35.75, *p* < 0.01, *η*_*p*_^2^ = .70) and context (*F*(1.15) = 5.50, *p* < 0.05, *η*_*p*_^2^ = .26). The interaction stimulus x context was non-significant, *F* < 1, indicating that the animals developed a hyperactivity conditioned compensatory response specific to the trained context: the animals display higher levels of activity in the absence of sucrose (test water) and are more tolerant to its effects (test sucrose).

### Experiment 3: dissociation of CPP and tolerance

During the pre-training test, the animals in the group control showed a preference score (for the less preferred context) of 0.33 (± 0.02 *SEM*), while the animals in group D-Ant showed a preference score of 0.33 (± 0.01 *SEM*). A one-way ANOVA showed that there were no differences between the groups, *F* < 1.

**Fig. 5:**
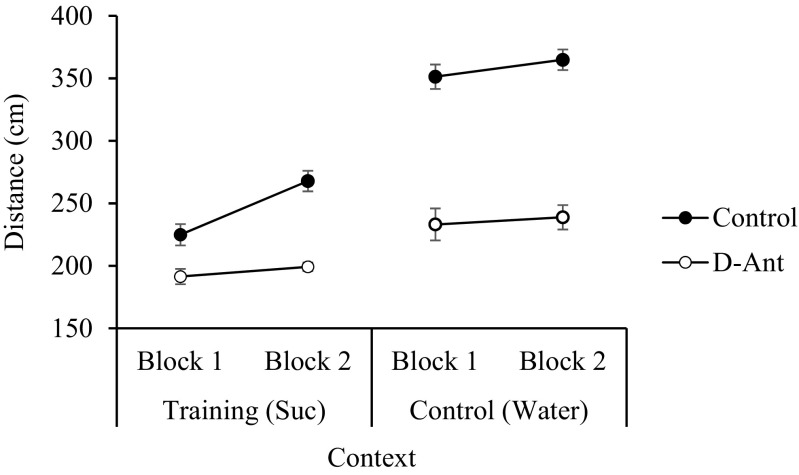
Mean distance (± *SEM*) covered by the animals of the two experimental groups (control and D-Ant) in the contexts were sucrose (training context) and water (control context) were presented over two blocks of 3 cycles of training during the training phase of experiment 3

The data of the training phase of the experiment are displayed in Fig. 5, which shows the activity of the two groups of the animals across two blocks of 3 cycles of training trials in the contexts associated with sucrose and water. The animals showed lower levels of activity in the presence of sucrose than in the presence of water. Additionally, the animals in the D-Ant group, exposed to the dopamine antagonist, showed lower levels of activity than the control group. A mixed ANOVA with one between-subject factor, group (control vs. D-Ant); two within-subject factors, stimulus (sucrose vs. water); and blocks (of cycles of training) showed a significant effect of group (*F*(1.30) = 160.45, *p* < 0.01, *η*_*p*_^2^ = .84); stimulus (*F*(1.30) = 165.05, *p* < 0.01, *η*_*p*_^2^ = .84); and blocks (*F*(1.30) = 6.36, *p* < 0.05, *η*_*p*_^2^ = .17). The interaction stimulus x group was also significant (*F*(1.30) = 35.89, *p* < 0.01, *η*_*p*_^2^ = .54). Further analyses carried out to assess the stimulus x group interaction showed that the stimulus factor was significant for the control group (*F*(1.15) = 184.46, *p* < 0.01, *η*_*p*_^2^ = .92) and for the D-Ant group (*F*(1.15) = 22.60, *p* < 0.01, *η*_*p*_^2^ = .60).

The blocks’ significant effect would suggest a general increase in the levels of activity throughout the training trials. However, as can be seen in Fig. 5, it is the increase of activity in the control group in the presence of sucrose that mainly contributes to the general increase of activity across the training cycles. Although the triple interaction stimulus x group x blocks was not significant (*F*(1.30) = 2.20, *p* = 0.10), we analysed the effect of the exposure to sucrose and water on the activity across the blocks of trials for the two experimental groups separately. A within-subject ANOVA with stimulus (sucrose vs. water) and blocks carried out on the data of the D-Ant group showed a significant effect of stimulus (*F*(1.15) = 22.60, *p* < 0.01, *η*_*p*_^2^ = .60); however, neither the block factor nor the stimulus x block interaction was significant, *F*s < 1. The same analysis on the data of the group control showed a significant effect of stimulus (*F*(1.15) = 184.79, *p* < 0.01, *η*_*p*_^2^ = .92) and blocks (*F*(1.15) = 11.00, *p* < 0.01, *η*_*p*_^2^ = .42) and a close to significant stimulus x block interaction (*F*(1.15) = 3.87, *p* = 0.06, *η*_*p*_^2^ = .20). Analysis of the main effects showed a significant increase in the levels of activity in the presence of sucrose across the two blocks of cycles of training (*F*(1.15) = 13.49, *p* < 0.01, *η*_*p*_^2^ = .47), but not in the presence of water (*F*(1.15) = 1.52, *p* = 0.23); this suggests that the animals in the control group developed tolerance to the effects of the sucrose.

**Fig. 6:**
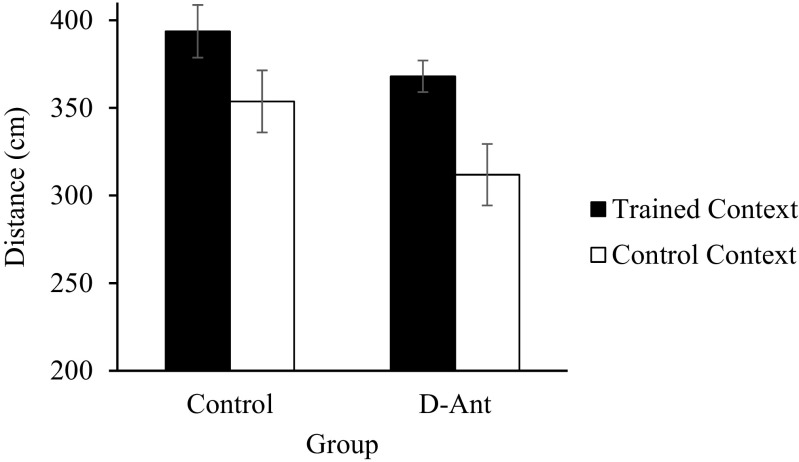
Mean distance (± *SEM*) covered by the animals in the groups control and D-Ant in the trained and the control contexts in the presence of water during the conditioned compensatory response test of experiment 3

The data of the test water (assessing the hyperactivity conditioned compensatory response) are displayed in Fig. 6. The animals showed a hyperactivity conditioned response in the trained context (associated with sucrose during training) compared to the control context both in the groups control and D-Ant. A mixed ANOVA with a between-subject factor, group (control vs. D-Ant), and a within-subject factor, context (trained vs. control), showed a significant effects of group (*F*(1.30) = 4.198, *p* < 0.05, *η*_*p*_^2^ = .12) and context (*F*(1.30) = 11.96, p < 0.01, *η*_*p*_^*2*^ = .28); the interaction group x context was not significant *F* < 1.

**Fig. 7:**
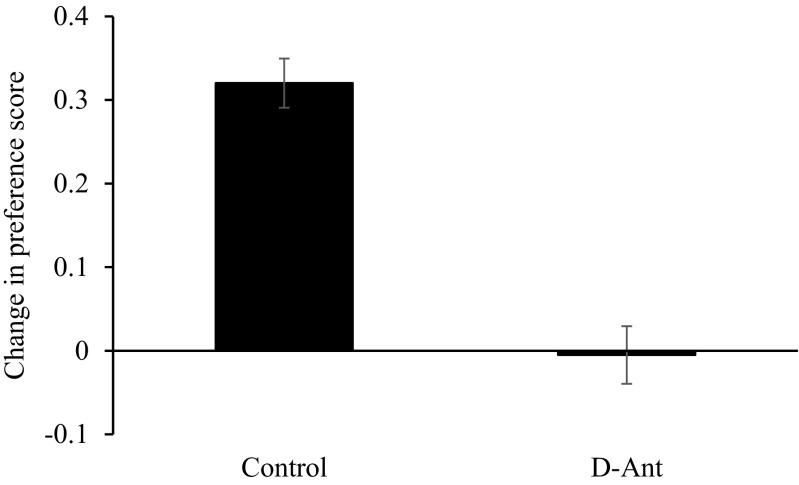
Mean change in preference score (± *SEM*) to the target context in the control and the D-And groups during the CPP test of experiment 3

The data for the CPP test (change in preference score for the initially non-preferred context) are displayed in Fig. 7. The animals in the control group showed strong evidence of CPP, whereas the animals in the D-Ant group did not show any change in preference. A one-way ANOVA confirmed this impression (*F*(1.30) = 51.57, *p* < 0.01).

## Discussion

The present research aimed to assess the nature of the mechanisms that modulate addictive-like behaviours in the planarian. Following exposure to sucrose and water in distinctive contexts, the planarian showed a conditioned place preference (CPP) response. Previous attempts to assess sucrose-mediated CPP in planarian assessed the aftereffects of sucrose rather than long-term CPP and did not equate the experience with the two surfaces—rewarded and non-rewarded (Zhang et al. 2013). In the present experiment, the animals were equally exposed to the two surfaces during training and were tested 24 h after the last training cycle, thus demonstrating genuine long-term CPP.

It has been often suggested that, in the planarian, dopamine plays an important role in movement control (e.g. Butarelli et al. 2000) as well as in reward-related learning. In our experiments, the animals treated with dopamine D1 antagonist did not develop a CPP response.^3^ The present results confirm that the dopamine reward system mediates the establishment of appetitive Pavlovian conditioned responses like CPP in the planarian.

Our study also showed that the sucrose-stimulated CPP extinguishes in the absence of the reward and can be reinstated by exposure to the rewarding agent. This confirms that CPP can be characterised as a standard Pavlovian conditioned response in the *Planaria*. Previous attempts to demonstrate reinstatement in the planarian confounded it with re-conditioning: in the study by Amaning-Kwarteng et al. (2017), for example, following extinction, the animals were exposed to the rewarding agent, cocaine, in the cocaine-paired surface. In the present study, however, the animals were exposed to the rewarding agent, sucrose, in a surface different to the ones used during the training phase of the experiment. Exposure to the rewarding agent per se could not, therefore, strengthen the association between the target context and the sucrose (the conditioned and the unconditioned stimuli  in Pavlovian terminology). During the test, however, a clear CPP response was observed suggesting that exposure to the US promotes retrieval of the excitatory association established during the acquisition in detriment of the more recently acquired inhibitory association that develops during extinction; a genuine demonstration of the reinstatement effect known to mediate relapse after periods of abstinence (Bouton 2004; Bouton and King 1983; Shaham et al. 2003).

Repeated exposure to sucrose results in the development of tolerance to its effects: the slowing response initially observed to sucrose weakens with experience. This tolerance was proven to be context dependent: the results of the test water show that the animals developed a hyperactivity conditioned compensatory response selectively controlled by the contextual cues associated with sucrose (Siegel 1975). Moreover, the results of the test sucrose show that the animals are more tolerant to the effects of sucrose (reduced activity) in the trained context; sucrose was more effective in reducing the activity of the animals in the control context in which it had never been presented before.

The most relevant outcome of our research is the dissociation between the learning mechanisms leading to CPP and the development of drug tolerance by using a dopamine antagonist. CPP makes the context in which the effects of a drug are experienced attractive. This involves the establishment of an appetitive Pavlovian response mediated by the dopamine reward system. On the contrary, the development of tolerance can make the same context aversive: exposure to the context elicits a conditioned compensatory response (in our model, hyperactivity) which induces an imbalance in the organism. Regular consumers of a drug or addicts can restore the balance by taking the drug. Failing to take it, withdrawal symptoms characterised by distress and drug-seeking behaviour would be observed.

The treatment of drug addiction has to address these two components, CPP and the tolerance-related conditioned compensatory response: on the one hand, extinction of the CPP would prevent the individual to self-expose to the situations that are likely to result in distress and consumption. On the other hand, treatment needs to address the distressing conditioned compensatory response that leads to drug seeking and consumption. Our CPP and tolerance procedures in planarian are an ideal model for the development of pre-clinical behavioural (based upon extinction and counter-conditioning) and pharmacological protocols for the treatment of substance abuse and addiction.

## Footnotes

1 Previous research on sucrose-mediated CPP in *Planaria* has shown that treatment with D1 and D2 antagonists (1 μM SCH-23390 and 1 μM sulpiride) effectively disrupt the development but not the expression of a previously acquired CPP response compared to non-treated control groups (Zhang et al. 2013). In the absence of differences between the effects of D1 and D2 antagonists, we decided to focus on the effect of 1-μM SCH-23390 on the development of CPP.

2 Preliminary experiments in our laboratory compared the development of CPP in the presence of different concentrations of sucrose (for example, 1 and 10% and a control condition treated with plain water). The results showed that although the animals show a shift in preferences in the group 1%, it is difficult to obtain significant differences with the control group. This is due to the fact that the animals exposed to water in the preferred and non-preferred contexts develop habituation to the non-preferred context and tend to spend around 50% of the time in each surface during the post-training tests. This slight change in preferences in the control condition reduces the sensitivity of the test to detect a significant change of preferences with a mild reward, 1% sucrose. A stronger reward, 10% sucrose, proved to be more reliable in producing significant CPP and that is why we have used the 10% sucrose solution in all the experiments reported here.

3 In preliminary experiments using the CPP procedure, we compared the effects of the 1 μM SCH-23390 with a control water-only group. These experiments showed that pairing one of the surfaces with the SCH-23390 alone did not result in significant preference changes, confirming that the action of the D1 antagonist is the prevention of CPP rather than the development of, for example, conditioned place aversion.
